# Association of a Callback Program With Emergency Department Revisit Rates Among Patients Seeking Emergency Care

**DOI:** 10.1001/jamanetworkopen.2022.13154

**Published:** 2022-05-20

**Authors:** Scott Fruhan, Corey B. Bills

**Affiliations:** 1 Department of Emergency Medicine, University of California, San Francisco; 2 Department of Emergency Medicine, School of Medicine, University of Colorado, Aurora

## Abstract

**Importance:**

The emergency department (ED) discharge process often involves haste and poor communication.

**Objectives:**

To assess the association of an automated telephone call 2 days after ED discharge with the likelihood of an unplanned ED revisit at both 72 hours and 7 days after the index visit and with perceived care metrics measured at 14 days.

**Design, Setting, and Participants:**

This prospective nonrandomized clinical trial was conducted at a single academically affiliated county emergency department among 8110 patients presenting to and discharged from the ED during a 10-week period from June 25 to August 30, 2018. Initial statistical analysis was performed from February 1 to November 30, 2020, with additional analyses performed from March 1 to 16, 2022.

**Interventions:**

Participants were allocated in a nonrandom fashion to 1 of 2 groups: patients who received an automated telephone call 2 days after discharge were compared with patients who received no call 2 days after discharge. All patients received a telephone questionnaire at 14 days to assess secondary outcome measures.

**Main Outcomes and Measures:**

The primary outcome was a return visit to the ED at 7 days. Secondary outcomes included a return visit to the ED within 72 hours, a return visit to the ED within 7 days resulting in hospital admission, and patient-reported perceptions of their care measured by 4 questions related to quality metrics assessed at 14 days. A secondary analysis compared patients who actually responded to the initial call at 2 days with all nonresponders, regardless of whether they received a call. Analyses were made on an intention-to-treat basis.

**Results:**

More than 15 000 patients were seen in the ED during the 10-week study period, and 10 948 were discharged. A total of 8110 patient encounters (4460 male patients [55.0%]; 3313 Hispanic patients [40.9%]; mean [SD] age, 40.5 [19.4] years) were enrolled. A total of 2958 patients (36.5%) received an automated telephone call at 2 days after discharge, while 5152 (63.5%) received no call. Rates of ED return within 7 days of the initial index visit were significantly lower among those who received a call at 2 days than those who did not receive a call (224 of 2958 [7.6%] vs 533 of 5152 [10.3%]; *P* < .001). Patients who received a 2-day call were more likely than those who did not receive a call to have followed up with a health care clinician (67.9% [374 of 551] vs 66.3% [604 of 911]), understood their health issues (77.5% [490 of 632] vs 74.9% [780 of 1042]), and have received their discharge medications (87.0% [507 of 583] vs 83.6% [793 of 949]), although none of these differences between the 2 groups were statistically significant.

**Conclusions and Relevance:**

A telephone call to patients 2 days after discharge from the ED was associated with decreased ED use at 7 days after the index visit and may have been associated with marginal improvements in measured quality of care metrics.

## Key Points

**Question:**

Is a follow-up telephone call to patients 2 days after discharge from the emergency department (ED) associated with decreased ED use within 7 days after the index visit?

**Findings:**

This nonrandomized clinical trial of 8110 patients found that the rate of return to the ED within 7 days of the index visit was significantly lower for patients who received a follow-up call at 2 days.

**Meaning:**

This study suggests that directed telephone calls may be associated with decreased ED use among a select group of patients.

## Teaser

This nonrandomized clinical trial assesses the association of an automated telephone call 2 days after emergency department discharge with the likelihood of an unplanned emergency department revisit at both 72 hours and 7 days after the index visit and with perceived care metrics measured at 14 days.

## Introduction

The emergency department (ED) discharge process necessitates communication of complex information in a clear and concise way.^1^ However, information is often explained quickly and may be difficult for patients to remember. In 1 study, in which 140 adult English-speaking patients or caretakers were interviewed after ED discharge, only 13% understood all 4 of the major domains of discharge instructions: diagnosis and cause, ED-based care, post-ED care, and instructions on when to return to the ED.^2^ Inadequate or unclear communication during the ED medical encounter has been associated with decreased patient satisfaction, worse clinical outcomes, and unintended return visits to clinical care.^3,4,5^

Return visits to the ED are a strain on health systems—with substantially increased costs, patient volumes, treatment delays, and mortality.^6,7,8^ Reductions in ED revisits represent an opportunity to improve care and have been used as a quality metric for some time.^9^ Automated telephone callback programs are a common strategy for improving the quality of ED care, patient understanding, medication adherence, and discharge follow-up, as well as for reducing unneeded or repeated ED visits and potential hospital admission. However, most studies are observational and focus on patient satisfaction outcomes alone. High-quality, peer-reviewed evidence about ED visit reduction programs, including those with telephone-based interventions, remains limited, to our knowledge.^10^ In this study, we aim to assess the association of a 2-day postdischarge telephone call with metrics of perceived quality of care measured at 14 days and also with rates of ED return at both 72 hours and 7 days after the index visit, as well as subsequent return visits resulting in hospital admissions.

## Methods

We performed a prospective nonrandomized pragmatic clinical trial aimed at improving the discharge process and the rate of ED return within 7 days. Patients were discharged from a single large, urban, academically affiliated county ED in the United States during a 10-week period, from June 25 to August 30, 2018. In this academic training center, patients are seen by care teams consisting of interns, residents, fellows, and advanced practice clinicians (eg, physician assistants and nurse practitioners) and staffed by an attending physician in emergency medicine. This study was reviewed by the University of California, San Francisco institutional review board and was granted a not-human-participants determination based on the involvement of quality improvement activities. Per institutional review board determination, specific consent for enrollment was waived because the project was determined to be program evaluation and quality improvement activities. We referenced and adhered to the International Society for Pharmacoeconomics and Outcomes Research (ISPOR) reporting guideline for nonrandomized studies during the process.^11,12^

### Participants

Patients, regardless of age, were included if they were seen by an emergency care clinician and subsequently discharged from the ED, left against medical advice, or left prior to formal discharge after the conclusion of their ED care. Patients without a documented US-based 10-digit telephone number in the medical record were excluded from the study. Patients admitted to the hospital, transferred to another inpatient facility, discharged to jail, or who left prior to being seen or prior to completion of triage were also excluded from the study. Demographic data were collected for all participants and included age, sex, race and ethnicity, marital status, preferred language, and homelessness. Race and ethnicity were defined neither by the investigator nor participants but by predetermined ED registration categories. The category of “other” in race and ethnicity is self-reported and defined as not representative of the other categories provided. Race and ethnicity constituted 1 of several categories used to assess initial similarity between the 2 study groups and was not part of the final analysis. As part of the standard discharge process, patients were given documentation informing them to expect at least 1 follow-up telephone call to ask about their care. The decision to participate in telephone calls was at the patient’s discretion and was not associated with the ED-based clinical care.

### Intervention: 2 Days After Discharge

On a nonrandom subset of days amounting to approximately one-third of the study period, the callback program enrolled all eligible patients discharged from the ED. These patients or their surrogates (in the case of patients <18 years of age) received an automated telephone call, via a third-party contractor, 2 days after their ED visit. Calls were conducted in English, Spanish, and Cantonese. Individual clinicians were not aware of which patients would receive a 2-day call. The initial rollout and feasibility of the program led to a natural experimental design, wherein only some patients discharged from the ED during the study period received a call.

The automated call script (eMethods in the Supplement) focused foremost on determining whether patients wished to receive a callback from a clinician to clarify any part of their care plan. If a callback was requested, an advanced practice clinician made a same-day telephone call. In total, up to 3 attempts were made on the same day to reach each patient.

Before concluding, the 2-day automated call also prompted patients to answer a series of questions regarding their care: “Do you have questions about the discharge instructions you were given?” “Do you have questions about your follow-up plan?” “Do you have questions about the medications prescribed or recommended to you in the ED?” “Do you have a different type of question?” In each instance, patients were asked to automate their response by use of their telephone’s touch-tone buttons. No smartphone was necessary to complete the tasks.

### Patient-Reported Quality Metrics: 14 Days After Discharge

At 14 days, all patients discharged from the ED who met the inclusion criteria, including those who received calls at 2 days and those who did not receive a call, received an automated telephone questionnaire via the same third-party contractor (eMethods in the Supplement). The questions were based on prevalidated metrics of discharge success as described by the Agency for Healthcare Research and Quality and the Emergency Department Patient Experience of Care.^13^ The following questions were included: “Did you understand what your main health problem was during your visit to the emergency department?” “If a doctor or nurse in the emergency department told you to take medicines at home that you had not been taking before, were you able to get these medicines?” “Have you visited your healthcare provider or made an appointment to visit your healthcare provider since leaving the emergency department?” “Would you recommend this emergency department to your friends and family?”

### Outcomes

The primary outcome was a return visit to the ED within 7 days of the index visit. Secondary outcomes included a return visit to the ED within 72 hours, a return visit to the ED resulting in admission to the hospital within 7 days of the index visit, and the answers from 4 questions on measures of discharge success from the automated telephone questionnaire conducted at 14 days.

### Patient and Public Involvement

There was no patient or public involvement in the design or undertaking of this study. This project was part of larger health facility quality improvement efforts and informed by previous data on patient quality. Patients were not initially required to provide consent for enrollment on discharge. However, as part of the standard discharge process, patients were given documentation informing them to expect at least 1 follow-up telephone call asking about their care.

### Statistical Analysis

Initial statistical analysis was performed from February 1 to November 30, 2020, with additional analyses performed March 1 to 16, 2022. The sample size was based on a preintervention return rate at 7 days of 12%, with an anticipated absolute reduction of 3% in the intervention group. Assuming a 1:2 ratio of experimental to control patients, we anticipated a need for 1248 patients in the intervention group and 2497 patients in the control group, based on 0.8 as the probability (power) and .05 as type I error probability. Initial significance was set to *P* < .05 in 2-sided tests and conservatively modified to *P* < .01 in the 2 secondary analyses given the potential for type I error.

Statistical analysis was performed via Stata, version 14 (StataCorp LLC). Demographic data of enrolled patients were presented as numbers and percentages. Baseline data, stratified by whether patients were included in the 2-day call group, were assessed for distinctions between the 2 groups given the nonrandom nature of enrollment. Patient responses to questions at 2 and 14 days after discharge are also provided as numbers and percentages. Comparison of outcomes between baseline demographic characteristics and outcomes between the 2 groups, those included in the 2-day call group and those not included, were made using the Mantel-Haenszel summary χ^2^ test for categorical variables and the *t* test for continuous variables. Initial analyses were made on an intention-to-treat basis, in which the 2-day automated telephone call soliciting a patient’s desire for further contact constituted the offer of treatment.

Because enrollment in the group that received the 2-day call was not random and may not have been equally distributed across all days, we also performed a mixed-effect logistic regression model with day as a random effect. A secondary subgroup exploratory analysis was also performed, comparing patients who responded to the initial call at 2 days with all those not reached, regardless of whether they received a call attempt. Comparison of outcomes was performed by the χ^2^ test. This analysis was to test the presumption that patients who were called and successfully responded at 2 days may have had characteristics different from patients who were called but not reached.

Last, given the high baseline rate of ED use within this study population and the potential difference between the 2 study groups in numbers of patients with high rates of prior ED use, we conducted another exploratory analysis adjusting for patients with prior high rates of ED use. We defined high use as 3 or more ED visits within the 180 days prior to the index visit. The decision to perform separate adjusted logistic regression models for those with high and low rates of prior ED use and for the association of a 2-day call with 7-day revisits within the 2 groups was supported through significant interaction tests.

## Results

During the 10-week study period, there were 15 668 unique ED patient encounters (Figure); 10 500 patients were discharged, 186 left against medical advice, and 262 left prior to formal discharge, while 4720 were admitted, transferred, or left without being seen or triaged. Of the 10 948 eligible patient encounters, 2838 patients either did not have or did not report a telephone number for a callback, resulting in 8110 patients (74.1%; 4460 male patients [55.0%]; mean [SD] age, 40.5 [19.4] years) being enrolled in the study (Table 1). Patients were predominantly Hispanic (3313 [40.9%]) and African American (1794 [22.1%]). The initial preferred language among patients was English (6072 [74.9%]), and a large proportion of patients were experiencing homelessness or marginal housing (1053 [13.0%]).

**Figure.**
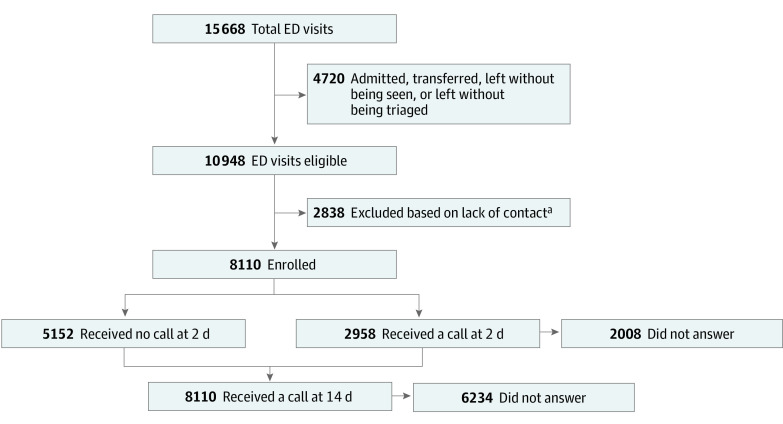
Flow Diagram of Patient Study Cohort ED indicates emergency department. ^a^Patients either did not have or did not report a telephone number for inclusion in the medical record for a callback.

**Table 1.** Baseline Characteristics of Index Presentations Over the 10-Week Study Period

Characteristic	Patients, No. (%)			*P* value
	Total enrolled (N = 8110)	2-d Call (n = 2958 [36.5%])	No 2-d call (n = 5152 [63.5%])	
Sex				
Female	3650 (45.0)	1304 (44.1)	2346 (45.5)	.21
Male	4460 (55.0)	1654 (55.9)	2806 (54.5)	
Age, mean (SD), y	40.5 (19.4)	39.5 (19.6)	41.2 (19.2)	<.001
Race and ethnicity				
African American (Black)	1794 (22.1)	616 (20.8)	1178 (22.9)	.04
American Indian or Alaska Native	49 (0.6)	19 (0.6)	30 (0.6)	
Asian	1032 (12.7)	419 (14.2)	613 (11.9)	
Hispanic	3313 (40.9)	1223 (41.3)	2090 (40.6)	
Native Hawaiian or Pacific Islander	110 (1.4)	32 (1.1)	78 (1.5)	
White	1583 (19.5)	570 (19.3)	1013 (19.7)	
Other^a^	197 (2.4)	69 (2.3)	128 (2.5)	
Unknown or declined to specify	32 (0.4)	10 (0.3)	22 (0.4)	
Preferred primary language				
Cantonese or Mandarin	292 (3.6)	115 (3.9)	177 (3.4)	.19
English	6072 (74.9)	2180 (73.7)	3892 (75.5)	
Spanish	1527 (18.8)	587 (19.8)	940 (18.2)	
Other	219 (2.7)	76 (2.6)	143 (2.8)	
Marital status				
Divorced or widowed	843 (10.4)	280 (9.5)	562 (10.9)	.11
Married or domestic partner	1484 (18.3)	567 (19.2)	917 (17.8)	
Single	5429 (66.9)	1978 (66.9)	3451 (67.0)	
Unknown, other, or not applicable	354 (4.4)	133 (4.5)	221 (4.3)	
Homeless				
Yes	1053 (13.0)	342 (11.6)	711 (13.8)	.004
No	7057 (87.0)	2616 (88.4)	4441 (86.2)	
Insurance				
Medicaid	4954 (61.1)	1808 (61.1)	3146 (61.1)	.31
Medicare	680 (8.4)	230 (7.8)	450 (8.7)	
Private	1444 (17.8)	553 (18.7)	891 (17.3)	
Other public or subsidized insurance	533 (6.6)	194 (6.6)	339 (6.6)	
None or other	499 (6.2)	173 (5.8)	326 (6.3)	
No. of visits in past 180 d				
0	6340 (78.2)	2125 (71.8)	3515 (68.2)	<.001
1	1138 (14.0)	419 (14.2)	719 (14.0)	
2	541 (6.7)	198 (6.7)	343 (6.7)	
3	240 (3.0)	79 (2.7)	161 (3.1)	
≥4	551 (6.8)	137 (4.6)	414 (8.0)	
Arrival by ambulance				
Yes	1736 (21.4)	663 (22.4)	1073 (20.8)	.09
No	6374 (78.6)	2295 (77.6)	4079 (79.2)	
Length of stay, mean (SD), min	278 (173)	268 (168)	283 (175)	<.001
Arrival time of day				
Morning (7 am-3 pm)	3138 (38.7)	1135 (38.4)	2003 (38.9)	.88
Afternoon (3 pm-11 pm)	3395 (41.9)	1241 (42.0)	2154 (41.8)	
Night (11 pm-7 am)	1577 (19.5)	582 (19.7)	995 (19.3)	

^a^ Other is self-reported and defined as not representative of the 6 categories of race and ethnicity.

At 2 days after the index visit, 2958 patients (36.5%) received a callback (Table 2). The remaining patients (n = 5152 [63.5%]) did not receive a call at 2 days. Demographic characteristics between the 2 groups are presented with baseline significance testing (Table 1). Of the 2958 patients called at 2 days, 950 (32.1%) answered, and 328 of 757 patients (43.3%) requested a telephone call by a clinician (Table 2). All 328 patients received a callback, and 224 (68.3%) were successfully reached. Patients often had more than 1 question or concern regarding their medical care and discharge plans. Interventions were undertaken during 115 of these 224 calls (51.3%), including help with follow-up appointments (74 of 115 [64.3%]), reviewing and supporting help with getting medications (37 of 115 [32.2%]), questions regarding patients’ diagnosis and treatment (71 of 115 [61.7%]), and support with understanding discharge instructions and reasons to return to ED (60 of 115 [52.2%]).

**Table 2.** Responses to a 2-Day Outreach Call Among a Sample of Patients Discharged From a Large Urban Emergency Department

Characteristic	Patients who received a 2-d call, No. (%)
No./total No. of patients who received a call	2958/8110 (36.5)
Patients who answered (n = 2958)	
Yes	950 (32.1)
No	2008 (67.9)
Primary language used (n = 759)	
English	438 (57.7)
Spanish	275 (36.2)
Cantonese	46 (6.1)
Requested callback (n = 757)	
Yes	328 (43.3)
No	429 (56.7)
Had discharge questions (n = 274)	
Yes	127 (46.3)
No	147 (53.7)
Had medication questions (n = 251)	
Yes	74 (29.5)
No	177 (70.5)
Had follow-up plan questions (n = 240)	
Yes	152 (63.3)
No	88 (36.7)
Had another question or request (n = 228)	
Yes	112 (49.1)
No	116 (50.9)

At 14 days after the index ED visit, 8810 patient encounters resulted in an automated telephone survey attempt. Of those, 1876 patients (21.3%) were successfully reached, and 1438 of those successfully reached (76.7%) answered all of the questions. At 14 days, patients who had received a call at 2 days were more likely than those who did not receive a call to report understanding their discharge plan (490 of 632 [77.5%] vs 780 of 1042 [74.9%]), success in getting medications (507 of 583 [87.0%] vs 793 of 949 [83.6%]), and successful health care outpatient follow-up plans (374 of 551 [67.9%] vs 604 of 911 [66.3%]), but none of these differences were statistically significant (Table 3). When assessed for primary outcomes by univariable analysis, the rate of return to the ED was significantly lower among all those who received a call at 2 days (whether successfully reached or not) compared with those who did not receive a call, at both 72 hours (137 of 2958 [4.6%] vs 319 of 5152 [6.2%]; *P* = .003) and 7 days (224 of 2958 [7.6%] vs 533 of 5152 [10.3%]; *P* < .001) (Table 4). Enrollment was not equally distributed across all initial visit days, however. The unadjusted odds of returning within 72 hours and 7 days were, again, significantly lower in the group who received a call (72 hours: odds ratio [OR], 0.71 [95% CI, 0.60-0.83]; 7 days: OR, 0.74 [95% CI, 0.60-0.90]); after adjusting for day as a random effect, an even more negative association was found between the 2-day call and the odds of return to the ED at both 72 hours and 7 days (72 hours: OR, 0.58 [95% CI, 0.47-0.71]; 7 days: OR, 0.63 [95% CI, 0.48-0.82]). Owing to the high baseline rate of ED use within this study population, we sought to control for patients with prior high rates of ED use. When the outcomes were modeled to include the interaction of frequent ED use with the incidence of receiving a 2-day call, the interaction terms were found to be significant for both 72-hour (mean [SE] interaction term, –0.620 [0.265]; *P* = .02) and 7-day revisit rates (mean [SE] interaction term, –0.593 [0.214]; *P* = .006) (eTable 1 in the Supplement). Significantly more individuals were likely to be in the 2-day call group of the study if they had fewer than 3 visits in the previous 180 days (eTable 2 in the Supplement). In addition, having had 3 or more ED visits in the previous 180 days was independently associated with higher revisit rates at 7 days after discharge.

**Table 3.** Comparison of Patient Quality Outcomes at 14 Days Between Patients Who Received a Call at 2 Days and Those Who Did Not

Characteristic	Patients, No./total No. (%)		*P* value
	2-d Call	No 2-d call	
Has any concern			
Yes	284/666 (42.6)	487/1089 (44.7)	.40
No	382/666 (57.4)	602/1089 (55.3)	
Understands discharge plan			
No	142/632 (22.5)	262/1042 (25.1)	.22
Yes	490/632 (77.5)	780/1042 (74.9)	
Able to get medications			
No	76/583 (13.0)	156/949 (16.4)	.07
Yes, or told not to take	507/583 (87.0)	793/949 (83.6)	
Has a follow-up appointment			
Has not visited nor made	177/551 (32.1)	307/911 (33.7)	.54
Visited or made in future	374/551 (67.9)	604/911 (66.3)	
Recommend care to others			
No	49/541 (9.1)	80/897 (8.9)	.93
Yes	492/541 (90.9)	817/897 (91.1)	

**Table 4.** Comparison of Clinical Outcomes at 7 Days Between Patients Who Received a Call at 2 Days and Those Who Did Not

Characteristic	Patients, No. (%)		*P* value
	2-d Call (n = 2958)	No 2-d call (n = 5152)	
Revisit at 72 h			
Yes	137 (4.6)	319 (6.2)	.003
No	2821 (95.4)	4833 (93.8)	
Revisit at 7 d			
Yes	224 (7.6)	533 (10.3)	<.001
No	2734 (92.4)	4619 (89.7)	
Return visit resulting in admission			
Yes	31 (1.0)	74 (1.4)	.14
No	2927 (99.0)	5078 (98.6)	

A 2-day call was associated with significantly fewer return visits at both 72 hours and 7 days among those with 3 or more ED visits within the 180 days prior to the index visit (72 hours: OR, 0.50 [95% CI, 0.32-0.80]; 7 days: OR, 0.49 [95% CI, 0.34-0.71]). However, a 2-day call was not associated with a significant difference in ED revisits among those who had fewer than 3 visits in the 180 days prior to the index visit (72 hours: OR, 0.93 [95% CI, 0.74-1.18]; 7 days: OR, 0.88 [95% CI, 0.73-1.07]). A 2-day call had no association with hospital admissions, regardless of the number of visits a patient had within the preceding 180 days of the index visit (<3 visits: OR, 0.68 [95% CI, 0.43-1.09]; ≥3 visits: OR, 1.24 [95% CI, 0.46-3.29]).

Last, we provide a secondary ad hoc analysis comparing outcomes among individuals successfully reached at 2 days (n = 950) and those who were called but not reached (n = 2008) with those not called at 2 days (n = 5152). Primary outcomes were similar to those of the nonstratified analyses, with 1 addition: patients who were successfully reached, compared with those who were not called, were not only significantly less likely to return to the ED at 7 days (6.2% [59 of 950] vs 10.3% [533 of 5152]; *P* < .001) (eTables 3-5 in the Supplement), they were also significantly less likely to be admitted to the hospital within 7 days after ED discharge (0.4% [4 of 950] vs 1.4% [74 of 5152]; *P* = .007). In addition, the subgroup of patients who were successfully reached at 2 days resembled the larger nonstratified 2-day call group in demonstrating a similar nonsignificant trend of greater likelihood than their unreached counterparts to report favorable quality metrics, such as success in getting medications and in follow-up plans. However, those successfully reached at 2 days did report a statistically significant difference in understanding the care they received (18.0% [61 of 338] vs 25.7% [343 of 1336]; *P* = .003) (eTable 6 in the Supplement).

## Discussion

Although ED callback programs are in broad use across the US, existing data have been variable and underpowered in their ability to demonstrate improvement in patient care metrics.^14,15,16^ In our study, an automated call 2 days after an ED discharge, with optional ED clinician follow-up if requested, was associated with a decrease in both 72-hour and 7-day return visits to the ED. In addition, while there was no significant difference in return visits resulting in hospital admission among all patients receiving calls at 2 days, there was a significant difference if the caller was actually reached. Finally, the callback program may have been associated with marginal improvements in subjective quality of care metrics.

Managing the ever-increasing numbers of patient presentations is a major challenge for EDs across the United States.^17^ Regardless of the cause of frequent ED use, telephone callbacks have been proposed as 1 mechanism for enhancing the discharge process by increasing the patient’s understanding of the discharge process, by providing additional social support, and, ideally, by reducing return visits.^18,19^ Although callback programs are often regarded as a way to increase patient satisfaction,^20^ this study showed little difference in patient-reported metrics of quality of care, including whether patients would recommend the ED to others. The subset of patients reached by callback, however, was significantly more likely to report understanding the care they received.

Although the presence of a callback had no association with overall satisfaction rates, nearly one-third of the 950 patients called and reached at 2 days requested a callback. A substantial number of those were successfully contacted and given direct clinical guidance regarding their concerns. In all, 7.6% (224 of 2958) of those who initially received an automated call connected directly with a clinician. Using an automated callback initially may reduce the overall burden of post-ED follow-up and serve as a screening tool to target those at higher need and potentially higher risk of poor outcomes.

As with patient-reported care metrics, prior evidence on the effectiveness of callback programs in reducing ED use has similarly been variable.^10^ Our data show revisit rates at both 72 hours and 7 days to be significantly lower among those who received a telephone call at 2 days after discharge. These data suggest the potential for ED callback programs to improve multiple outcome measures, from simple ED revisits to revisits resulting in hospital admission.

### Limitations

This study has some limitations. Callback programs have significant limitations with regard to reaching patients, and the one used in this study is no exception.^16^ First, this is a single-center study with a unique patient population: predominantly urban and indigent, with a high rate of frequent health care use. As such, the results may not be generalizable to other settings.

Second, this study is limited by the design and the nonrandom nature of patient assignments. Although the 2 study groups were alike in many ways, some baseline characteristics differed significantly. We have provided statistical methods to account for the possible effects of unevenly distributed variables, such as high health care use. Although this was a nonrandomized study, there was no indication that physicians were aware of which patients would be assigned to which study group, and the possibility of this form of systematic bias remains low.

Third, we do not provide data on revisits to hospitals or health care facilities outside of the single center under study. As a result, this study, like many others in the field, likely underestimates the true revisit rate after the index visit.

Fourth, in this study, most of the patients who were discharged from the ED were not reached by telephone; just over 30% of patients were successfully reached for the intervention event at 2 days, and 21% were reached for secondary outcomes analysis at 14 days. Patients with significant social needs, especially those without a reliable telephone or housing, have been shown to use the ED more often and may be missed by this intervention.^21^ The exclusion of all patients without telephone numbers clearly constitutes a selection bias likely favoring a population with more social or economic stability, thereby limiting the generalizability of results. At the same time, a stratified analysis of those with prior high use of ED services noted that they were more likely influenced by a 2-day call. Among the small subset of patients successfully reached at 2 days, specific clinician actions may have resulted in additional, unmeasured patient support.

Fifth, this study is limited by the extent to which the primary outcome serves as a true measure of quality. Seven-day return visits are a crude and sometimes (in the case of appropriate response to discharge instructions) inappropriate method of determining emergency care quality. Return visits within 7 days may not measure overall patient risk^22^ and may miss some unintended return visits that occur after this period.^23^ Although many ED-based metrics of quality of care exist, all have challenges, and 7-day return visits have an established literature base around which to compare our findings.

## Conclusions

This study suggests that there is measurable value to automated ED callback programs with a clinician contact option. This was evidenced by (1) the percentage of patients requesting clinician support, (2) the statistically significant improvement in the broadly recognized metric of the ED revisit rate, and (3) a statistically significant improvement in both revisit hospital admissions and patient-reported understanding of care among those who were successfully reached at 2 days after the initial discharge.

## Notes

Supplement. **eMethods.** ED Callback Scripts at 48 Hours Postdischarge and 14 Days Postdischarge **eTable 1.** Interaction Test Between ED Frequent Use and a 2-Day Call for ED Revisits Within 72 Hours and 7 Days Postindex Visit **eTable 2.** Comparison of Rates of Prior ED Utilization by Study Group Enrollment and ED Revisits at 7 Days **eTable 3.** Comparison of Clinical Outcomes at 7 Days Between Those Who Were Called and Reached at 2 Days and Those Not Called at 2 Days **eTable 4.** Comparison of Clinical Outcomes at 7 Days Between Those Who Were Called but Not Reached at 2 Days and Those Not Called at 2 Days **eTable 5.** Comparison of Clinical Outcomes at 7 Days Between Those Who Were Called and Reached at 2 Days and Those Who Were Either Not Called or Called But Not Reached at 2 Days **eTable 6.** Comparison of Patient Quality Outcomes Measured 14 Days Postdischarge Between Patients Who Were Called and Reached at 2 Days and Those Who Were Either Not Called or Not Reached at 2 Days
