# Supplementary material for: Association of a Callback Program With Emergency Department Revisit Rates Among Patients Seeking Emergency Care
Source: JAMA Netw Open. 2022 May 20;5(5):e2213154. doi: 10.1001/jamanetworkopen.2022.13154 (PMC9123498; doi:10.1001/jamanetworkopen.2022.13154)
Supplement: Supplement. — eMethods. ED Callback Scripts at 48 Hours Postdischarge and 14 Days Postdischarge eTable 1. Interaction Test Between ED Frequent Use and a 2-Day Call for ED Revisits Within 72 Hours and 7 Days Postindex Visit eTable 2. Comparison of Rates of Prior ED Utilization by Study Group Enrollment and ED Revisits at 7 Days eTable 3. Comparison of Clinical Outcomes at 7 Days Between Those Who Were Called and Reached at 2 Days and Those Not Called at 2 Days eTable 4. Comparison of Clinical Outcomes at 7 Days Between Those Who Were Called but Not Reached at 2 Days and Those Not Called at 2 Days eTable 5. Comparison of Clinical Outcomes at 7 Days Between Those Who Were Called and Reached at 2 Days and Those Who Were Either Not Called or Called But Not Reached at 2 Days eTable 6. Comparison of Patient Quality Outcomes Measured 14 Days Postdischarge Between Patients Who Were Called and Reached at 2 Days and Those Who Were Either Not Called or Not Reached at 2 Days [file jamanetwopen-e2213154-s001.pdf]

Supplementary Online Content

Fruhan S, Bills CB. Association of a callback program with emergency department revisit rates among patients seeking emergency care. *JAMA Netw Open.* 2022;5(5):e2213154. doi:10.1001/jamanetworkopen.2022.13154

eMethods. ED Callback Scripts at 48 Hours Postdischarge and 14 Days Postdischarge
eTable 1. Interaction Test Between ED Frequent Use and a 2-Day Call for ED Revisits Within 72 Hours and 7 Days Postindex Visit
eTable 2. Comparison of Rates of Prior ED Utilization by Study Group Enrollment and ED Revisits at 7 Days
eTable 3. Comparison of Clinical Outcomes at 7 Days Between Those Who Were Called and Reached at 2 Days and Those Not Called at 2 Days
eTable 4. Comparison of Clinical Outcomes at 7 Days Between Those Who Were Called but Not Reached at 2 Days and Those Not Called at 2 Days
eTable 5. Comparison of Clinical Outcomes at 7 Days Between Those Who Were Called and Reached at 2 Days and Those Who Were Either Not Called or Called But Not Reached at 2 Days
eTable 6. Comparison of Patient Quality Outcomes Measured 14 Days Postdischarge Between Patients Who Were Called and Reached at 2 Days and Those Who Were Either Not Called or Not Reached at 2 Days

This supplementary material has been provided by the authors to give readers additional information about their work.

eMethods. ED Callback Scripts at 48 Hours Postdischarge and 14 Days Postdischarge

ED Voice Callback Script – 48 hours postdischarge

1. **Opening**: Hello, this is nurse practitioner [redacted] from the [redacted] Hospital Emergency Department with an important follow up call about your health and progress. This call should take less than 2 minutes.

2. **Language:** To continue in English, press 1. To continue in Spanish, press 2. To continue in Cantonese, press 3.
   - English – Ok, thanks.
   - Spanish – Ok, thanks.
   - Cantonese – Ok, thanks.

3. **Who Answered**: If you visited the [redacted] Hospital Emergency Department in the past few weeks, please press 1. If you take care of someone who recently went to the Emergency Department at [redacted] Hospital and you were at the visit with them, press 2. To have us call back at a later time, press 3. Or, if we have reached the wrong number, press 4.
   - Patient - OK, thanks.
   - Caregiver - OK, thanks. Please complete this call on behalf of the patient.
   - Call Later - OK, thanks. We'll call you back at a later time. Have a nice day. Goodbye.
   - Wrong Number - OK, we're sorry to have bothered you. Have a nice day, goodbye.

4. **Callback Request:** Your discharge plan is important for your recovery after leaving the emergency department. Would you like an emergency health care provider to call you back to address questions about your discharge instructions, new medications, or follow-up plan? If you would like a call back, please press 1. If you do not want a call back, press 2.
   - Call back – OK, we will have a member of the emergency department team call you back. This should happen within the next 24 hours. [ALERT]
   - No call back – OK, thanks for letting us know. You will not receive a phone call from a clinician. Please note that you may still receive a separate automated phone call within the next two weeks asking for your help in improving the service and quality of the care we deliver.

5. **Discharge instructions:** In order to assist the person calling you back, we need a little more information. Do you have questions about the discharge instructions you were given? If you have questions about the instructions, press 1. If you do not have questions, press 2.
   - Yes - OK, thanks.

© 2022 Fruhan S et al. *JAMA Network Open.*

- No – OK, thanks.

6. **Medications**: Do you have questions about the medications prescribed or recommended to you in the emergency department? If you have questions about these medications, please press 1. If you do not have questions, press 2.
    - Yes - OK, thanks.
    - No – OK, thanks.

7. **Follow Up Plan**: Do you have questions about your follow-up plan? If you have questions, please press 1. If you do not have questions, press 2.
    - Yes - OK, thanks.
    - No – OK, thanks.

8. **Other:** If you have a different type of question, or questions about more than one of the items listed above, please press 1. If not, press 2.

Disclaimer/Goodbye: This concludes our Emergency Department follow-up phone call. Please note, if you're having medical problems now, or if you experience medical problems after this call, please consult a medical provider, or go to your nearest emergency department, or call 911. Thank you for participating in this call, and thank you for your time. Goodbye.

Sorry message: Sorry, we didn't understand your answer. Could you please listen to the question again?
ED Voice Callback Script – 14 day postdischarge

Opening: Hello, this is nurse practitioner [redacted] from the [redacted] Hospital Emergency Department with an important follow up call about the service and quality of the care we deliver. This call should take about 5 minutes.

Language: To continue in English, press 1. To continue in Spanish, press 2. To continue in Cantonese, press 3.

- English – Ok, thanks.
- Spanish – Ok, thanks.
- Cantonese – Ok, thanks.

1. **Identification:** If you visited the [redacted] Hospital Emergency Department in the past few weeks, please press 1. If you take care of someone who recently went to the Emergency Department at [redacted] Hospital and you were at the visit with them, press 2. To have us call back at a later time, press 3. Or, if we have reached the wrong number, press 4.

- Patient - OK, thanks. Let's get started.
- Caregiver - OK, thanks. Please complete this call on behalf of the patient. Let's get started.

© 2022 Fruhan S et al. *JAMA Network Open.*

- Later time – OK, thanks. We'll call you back at a later time. Have a nice day. Goodbye.
- Wrong Number - OK, we're sorry to have bothered you. Have a nice day. Goodbye.

2. **General Understanding:** Did you understand what your main health problem was during your visit to the emergency department?

If you understood your health problem, please press 1. If you partially understood, press 2. If you did not understand, press 3.

- Definitely - OK, thanks.
- Somewhat - OK, thanks.
- Did not - OK, thanks.

3. **Medications:** If a doctor or nurse in the emergency department told you to take medicines at home that you had not been taking before, were you able to get these medicines?

If you were able to get the new medicines, please press 1. If you were not able to get the new medicines, press 2. If you were not told to take any new medicines, press 3.

- If able to get new medications: OK, thanks.
- If unable to get new medications: OK, thanks.
- If not told to take medications: OK, thanks.

4. **Follow up**: Have you visited your healthcare provider or made an appointment to visit your healthcare provider since leaving the emergency department?
If you have visited your healthcare provider, please press 1. If you have made an appointment, press 2. If you have **not** visited or made an appointment, press 3.

- Visited - OK, thanks.
- Made appointment - OK, thanks.
- Neither - OK, thanks.

5. Recommendation: This is our last question. Would you recommend this emergency department to your friends and family?

If you would **definitely** recommend it, press 1. If you would **probably** recommend it, press 2. If you would probably **not** recommend it, press 3. If you would **definitely not** recommend it, press 4.

- Definitely yes – OK, thanks.
- Probably yes – OK, thanks.
- Probably not -- OK, thanks.
- Definitely not – OK, thanks.

© 2022 Fruhan S et al. *JAMA Network Open.*

Disclaimer/Goodbye: This concludes our Emergency Department follow-up phone call. We are always working to improve the consistency and quality of our communication at [redacted] Hospital. Please note, if you're having medical problems now, or if you experience medical problems after this call, please consult a medical provider, or go to your nearest emergency department, or call 911. Thank you for participating in this call. Goodbye.

Sorry message: Sorry, we didn't understand your answer. Could you please listen to the question again?

Voicemail message: Hi, this is a follow up call from [redacted] Hospital Emergency Department to see how you are recovering at home. Please call us back from the same phone you received this message on to answer a few short questions about the quality of the care we deliver. Have a nice day. Goodbye!

Inbound Message: Hi, you have reached Nurse Practitioner [redacted] from the [redacted] Hospital Emergency Department, thank you for returning our call. We have a few questions for you to answer about the service and quality of the care we deliver. This call should take about 5 minutes.

Inbound message mismatch:
Hi, thank you for calling [redacted] Hospital Emergency Department. If you are calling us back after receiving a voicemail, please hang up and call us back from the same phone you received the voicemail.

© 2022 Fruhan S et al. *JAMA Network Open.*

eTable 1. Interaction Test Between ED Frequent Use and a 2-Day Call for ED Revisits Within 72 Hours and 7 Days Postindex Visit

	Interaction Term		
**Model**	**ED Frequent Use * 2 Day Call**	**SE**	**p-value**
Revisit at 72 hours	-0.620	0.265	0.020
Revisit at 7 days	-0.593	0.214	0.006

© 2022 Fruhan S et al. *JAMA Network Open.*

eTable 2. Comparison of Rates of Prior ED Utilization by Study Group Enrollment and ED Revisits at 7 Days

	**Enrolled N (%)**			**Revisit at 7 days N (%)**		
ED Utilization (Visits last 180d)	2 Day Call	No 2 Day Call	p-value	Yes	No	p-value
All	2958 (36.5%)	5152 (63.5%)		757(9.3%)	7353 (90.7%)	
0 - 2 visits	2742 (37.5%)	4577 (62.5%)	<0.001	520 (7.1%)	6799 (92.9%)	<0.001
≥ 3 Visits	216 (27.3%)	575 (72.7%)		237 (30.0%)	554 (70.0%)	

© 2022 Fruhan S et al. *JAMA Network Open.*

eTable 3. Comparison of Clinical Outcomes at 7 Days Between Those Who Were Called and Reached at 2 Days and Those Not Called at 2 Days

Characteristic	Called And Reached at 2 Days N (%) N= 950	No 2 Day Call N (%) N=5152	p-value
Revisit at 72 hours			0.024
Yes	41 (4.3)	319 (6.2)	
No	909 (95.7)	4833 (93.8)	
Revisit at 7 days			<0.0001
Yes	59 (6.2)	533 (10.3)	
No	891 (93.8)	4619 (89.7)	
Return visit resulting in admission			0.007*
Yes	4 (0.4)	74 (1.4)	
No	946 (99.6)	5078 (98.6)	

*Fishers exact

© 2022 Fruhan S et al. *JAMA Network Open.*

eTable 4. Comparison of Clinical Outcomes at 7 Days Between Those Who Were Called but Not Reached at 2 Days and Those Not Called at 2 Days

**Characteristic**	**Called But Not Reached at 2 Days N (%) N= 2008**	**No 2-Day Call N (%) N=5152**	**p-value**
Revisit at 72 hours			0.022
Yes	96 (4.8)	319 (6.2)	
No	1912 (95.2)	4833 (93.8)	
Revisit at 7 days			0.006
Yes	165 (8.2)	533 (10.3)	
No	1843 (91.8)	4619 (89.7)	
Return visit resulting in admission			0.768
Yes	27 (1.3)	74 (1.4)	
No	1981 (98.7)	5078 (98.6)	

© 2022 Fruhan S et al. *JAMA Network Open.*

eTable 5. Comparison of Clinical Outcomes at 7 Days Between Those Who Were Called and Reached at 2 Days and Those Who Were Either Not Called or Called But Not Reached at 2 Days

Characteristic	Reached at 2-Day Call<br>N (%)<br>N= 950	No 2-Day Call or Not Reached<br>N (%)<br>N=7160	p-value
Revisit at 72 hours			0.06
Yes	41 (4.3)	415 (5.8)	
No	909 (95.7)	6745 (94.2)	
Revisit at 7 days			<0.0001
Yes	59 (6.2)	698 (9.7)	
No	891 (93.8)	6462 (90.3)	
Return visit resulting in admission			0.009*
Yes	4 (0.4)	101 (1.4)	
No	946 (99.6)	7059 (98.6)	

*Fishers exact

© 2022 Fruhan S et al. *JAMA Network Open.*

eTable 6. Comparison of Patient Quality Outcomes Measured 14 Days Postdischarge Between Patients Who Were Called and Reached at 2 Days and Those Who Were Either Not Called or Not Reached at 2 Days

Characteristic	Reached at 2 Day Call N (%)	No 2-Day Call or Not Reached N (%)	p-value
Has any concern			0.076
Yes	142 (36.2)	629 (45.0)	
No	215 (54.8)	769 (55.0)	
Understands discharge plan			0.003
No	61 (18.0)	343 (25.7)	
Yes	277 (82.0)	993 (74.3)	
Able to get medications			0.218
No	40 (12.9)	192 (15.7)	
Yes or told not to take	270 (87.1)	1030 (84.3)	
Has a follow-up appointment			0.228
Has not visited nor made	88 (30.1)	396 (33.8)	
Visited or made in future	204 (69.9)	774 (66.2)	
Recommend care to others			0.452
No	29 (10.1)	100 (8.7)	
Yes	258 (89.9)	1051 (9.1)	

© 2022 Fruhan S et al. *JAMA Network Open.*
